# Methods and reference data for middle ear transfer functions

**DOI:** 10.1038/s41598-022-21245-w

**Published:** 2022-10-14

**Authors:** M. Koch, T. M. Eßinger, H. Maier, J. H. Sim, L. Ren, N. T. Greene, T. Zahnert, M. Neudert, M. Bornitz

**Affiliations:** 1 Faculty of Medicine Carl Gustav Carus, ERCD-Ear Research Center Dresden at the Department of Otorhinolaryngology Head and Neck Surgery, TU Dresden, Fetscherstraße 74, 01307 Dresden, Germany; 2 Department of Otolaryngology, Hannover Medical School, Carl-Neuberg-Str. 1a, 30625 Hannover, Germany; 3 Cluster of Excellence “Hearing4all”, Hannover, Germany; 4 Department of Otorhinolaryngology, Head and Neck Surgery, University Hospital Zurich, University of Zurich, Zurich, Switzerland; 5 Eye and ENT Hospital of Fudan University, Fenyang Road 83, Shanghai, 200031 China; 6 Department of Otolaryngology-Head and Neck Surgery, University of Colorado School of Medicine, Aurora, CO USA

**Keywords:** Sensory systems, Translational research

## Abstract

Human temporal bone specimens are used in experiments measuring the sound transfer of the middle ear, which is the standard method used in the development of active and passive middle ear implants. Statistical analyses of these experiments usually require that the TB samples are representative of the population of non-pathological middle ears. Specifically, this means that the specimens must be mechanically well-characterized. We present an in-depth statistical analysis of 478 data sets of middle ear transfer functions (METFs) from different laboratories. The data sets are preprocessed and various contributions to the variance of the data are evaluated. We then derive a statistical range as a reference against which individual METF measurements may be validated. The range is calculated as the two-sided 95% tolerance interval at audiological frequencies. In addition, the mean and 95% confidence interval of the mean are given as references for assessing the validity of a sample group. Finally, we provide a suggested procedure for measuring METFs using the methods described herein.

## Introduction

### Background

Experimental studies using human cadaveric temporal bone (TB) specimens are an important tool for the understanding of middle ear mechanics and the development of new surgical procedures, diagnostics, or devices for hearing rehabilitation. Transferring the insights gained by such studies into real-world applications, however, requires classifying the mechanical properties of the samples used in relation to the population. An important characteristic here is the sound transfer from the tympanic membrane (TM) through the ossicular chain (OC) into the inner ear, which for non-pathological human middle ears and sound pressures of up to about 124 dB SPL or below can be represented with reasonable accuracy by a one-dimensional, linear time-invariant model system^1,2^. System theory states that such systems are characterized in the frequency domain by their transfer function^3^, and the characteristic function here is commonly called the middle ear transfer function (METF). The METF can be defined as the ratio

1 $$METF=\frac{Y(\omega )}{X(\omega )}$$

of one input $X(\omega )$ (sound pressure at the TM) and one output $Y(\omega )$ (sound pressure in the cochlea). Due to eigenfrequencies of the middle ear and adjacent structures, the METF has a characteristic shape, with a resonance at about 1 kHz and roll-off beyond this point^4^. Actual sound pressure measurements inside the cochlea can be challenging, so other signals are often used. A common procedure, which is also the focus of this study, is to measure the displacement or velocity of the stapes footplate (SFP) with a laser Doppler vibrometer (LDV)^5^, and to derive the sought-after sound pressure from these signals. Note that this indirect approach may introduce additional error. For instance, single point LDV measurements may not capture higher order modes of footplate movement (i.e. rocking, tilting or processing movement), which especially at higher frequencies ($f\ge 2 \mathrm{kHz}$) have a non-negligible contribution to total movement of the stapes^6,7^. Nevertheless, the simplicity of the application has led to this method becoming one of the most common measurement methods for determining METF worldwide.

Around the turn of the millennium, measurements of sound transfer in temporal bone specimens using LDV were becoming more widespread, in part due to advances in implantable hearing devices. Quantifying the normalized output of such devices in ex vivo experiments was the goal of Rosowski et al., whose important work led to a standardized procedure adopted as “ASTM F2504_05” by the American Society for Testing Materials^8^. This standard, which in accordance with general convention within the field of middle ear mechanics we shall simply call “ASTM standard”, gives a range of values for the (unaided) METF when measured using LDV, and states “Qualification Criteria” for temporal bones acceptable for further testing. These have since been widely used as validation in publications worldwide covering a range of topics in middle ear sound transfer, such as passive prostheses or reconstructive surgical techniques. However, it soon became apparent that a large number of TBs are ruled invalid by strict application of the original criteria. This was countered by modifying the criteria so as to allow for a more lenient validation process^9^. The cause for the discrepancy was not addressed until a paper published by Morse^10^, who showed that the statistical methods used in the ASTM standard are actually not well suited to derive the desired validation range for individual measurements.

The range given in the ASTM standard was calculated as the 95% confidence interval (CI) for the mean of means from 13 studies. This implies that it does not have a direct connection to the individual measurements that those means were calculated from. It could potentially be used as an estimate of where the mean of a new study is situated in comparison, but not for validation of individual temporal bones as has been practiced. Furthermore, the CI is proportional to the standard error of the mean and therefore to the inverse square root of the sample size ${n}^{-\frac{1}{2}}$, which means that the interval is smaller for a larger sample size. It is therefore an inappropriate measure for the desired purpose from the outset. That the so-called “modified criteria” of the ASTM standard have worked as well as they did in validating individual specimens is due to the fact that the ASTM mean is actually rather accurate and the modification of the width of the intervals is based on expert intuition and experience. However, TBs are a rare commodity and the ethics involved demand responsible handling and use. Therefore, the importance of correctly and objectively categorizing every single TB cannot be overstated. We have recently proposed a method using a tolerance interval (TI)^11^ of a reference group of METF data as comparison^12^. At the time, we used a data set from our own lab only to create the reference. In order for the method to be more generally applicable, we now present a multicenter evaluation of individual METF measurements. A total of 478 frequency responses measured in intact TBs from five labs in China, Germany, USA and Switzerland were available for this study.

### Aims of the study

Our main goal is to find a statistical range which may be used in the validation of METF measurements using sound pressure at the eardrum as the input signal and stapes footplate velocity (SFV) or displacement as the output signal, the latter measured using a single point of measurement near the center of the SFP. This range should be similar in practical application to the ASTM-range but it should be based on more representative statistics so as to be more reliable. We also aim to include all necessary materials and documentation so that the calculations described here can be extended with additional data or applied to other measurement methods.

**Figure 1:**
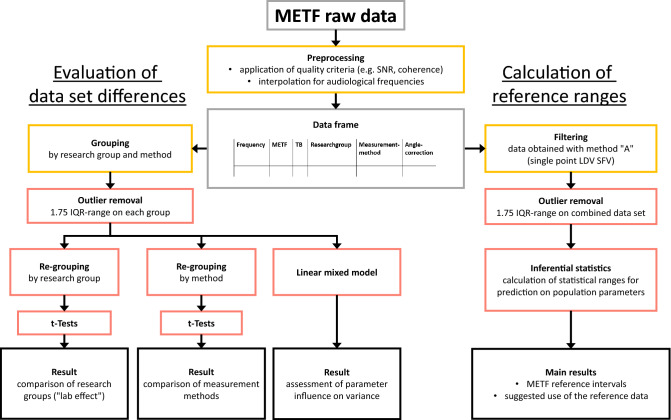
Outline of the study. Corresponding sections in the manuscript are color-coded: Yellow boxes (top) and red boxes (middle) refer to “Methods and Material”, subsections “Grouping and pre-processing of the data” and “Statistical Analysis”, respectively. Black boxes refer to “Results” and “Discussion” sections.

The data available for the study includes METF data of several international research groups, acquired using different methods of measurement. Therefore, we will first discuss the differences between the data from these different groups and methods (left side of the graph in Fig. 1). We will then group comparable data sharing a common methodology, from which the validation range will be calculated (right side of the graph in Fig. 1). This two-branch analysis allows for a more in-depth understanding of the impact certain variables have on the outcome.

## Methods and material

### Experimental setup

All methods were carried out in accordance with relevant guidelines and regulations. The experimental protocols were approved by an institutional committee (Technische Universität Dresden, Ethikkommision an der TU Dresden, Fetscherstr. 74, 01307 Dresden). Informed consent was obtained from all subjects and/or their legal guardian(s). The TBs included in this study were either fresh and kept refrigerated in isotonic NaCl-solution or freshly frozen upon harvesting and subsequently defrosted immediately before the measurement. The following is a general description of preparation and setup pertaining to the most commonly used way of determining the METF in TB specimens^5,13^. We will be labeling this approach as method “A”. Laboratory specific details or deviations from the general description are given below (see “Laboratory specifics”).

Following basic preparation (removal of connective tissue and muscles), access to the middle ear is accomplished via mastoidectomy and subsequent posterior tympanotomy. The cartilage and skin parts of the external ear canal are removed, leaving only the bony part of the external ear canal. The tympanic segment of the facial nerve is removed partially or completely in order to allow line-of-sight to the SFP for the LDV measurements. Care is taken to leave the whole ossicular chain, the attached ligaments and muscles (*Musculus stapedius* and *Musculus tensor tympani*), the TM, and the inner ear intact. In the process, the middle ear is inspected by the surgeon for any abnormalities. Pathological mobility of the OC and any other issues pertaining to sound transmission (such as a damaged cochlea or TM) are noted if detected. Note that the methods for inspection may vary, commonly this includes visual and tactile inspection. Reflective foil or beads are then placed onto the SFP. LDV measurements are acquired at a single point near the center of the SFP. For accurate measurements, it is important to remove excess water droplets from the SFP to ensure a reliable LDV signal. At the same time, the TB should be kept moist for the duration of the experiment so as to avoid changes in sound transmission due to drying of tissue, which typically begin to occur after about 60 min of drying at room temperature^14^. For METF measurements of the intact OC, this is not an issue because these are done immediately upon commencement of the experiment and usually do not require more than an hour.

An earphone for sound excitation is inserted into the ear canal and tightly sealed with an earplug to avoid uncontrolled loss of sound pressure during measurements. Excitement sound pressure levels of about 94–100 dB SPL are used to minimize nonlinear transmission effects. The ear canal is sometimes replaced partially or completely by an artificial volume. The probe microphone is inserted so that the tip of the probe sits a few millimeters in front of the TM. There are different ways of achieving this placement (see laboratory specifics below), but when done correctly they can be assumed to be equivalent in outcome. This is because the METF is per definition normalized to input pressure, and the measured sound pressure in TB experiments is insensitive to exact microphone position^14^. The METF is calculated by analogy to Eq. (1) with the respective discrete signals ${Y}_{k}={a}_{k}\cdot {V}_{k}^{LDV}$ and ${X}_{k}={b}_{k}\cdot {V}_{k}^{Probe}$, where ${V}_{k}$ denotes the magnitude of the fast Fourier transform (FFT) of the voltage received, ${a}_{k}$ and ${b}_{k}$ are calibration factors and the index $k$ symbolizes discrete frequency. Note that due to anatomical restrictions, the laser beam is usually pointed at the SFP at an incidence angle $\Theta >0$ to the normal axis of the SFP plane. This means that the measurement potentially underestimates the actual motion magnitude, because ${d}_{measured}={d}_{actual}\cdot \mathrm{cos}(\theta )$, where $d$ is the magnitude of stapes displacement. Therefore $\Theta$ itself or a range for the angle is usually recorded along with the experimental data.

### Laboratory specifics

The routines outlined above represent the most common procedure for determining the METF in human TBs. However, comparable results could be produced in a number of ways. Because this study includes data from an international set of laboratories, the specific conditions and methods for measurements differ. Additional descriptions of the methods used by the different research groups can be found below. Basic information on the different setups is summarized in Table 1.

**Table 1:** Excluded data. Numbers are derived by applying the Tukey method with k = 1.75.

Data group	Raw data sample size	Excluded samples
CO, Method D	18	2
DR, Method A	82	6
HA, Method A	268	30
SH, Method A	9	0
ZU, Method A	42	5
ZU, Method B	23	3
ZU, Method C	36	0
All raw data with Method A	401	35

#### Technische Universität Dresden

The LDV used was an OLV1000 with an OLV700 sensor head (both Polytec GmbH, Germany). The LDV angle of incidence was not recorded but was documented to be $\Theta \le 30^\circ$. The reference microphone, an ER-7C (Etymotic Research Inc., USA), was inserted through a close-fitting hole drilled into the anterior side of and perpendicular to the ear canal, ending 2–3 mm in front of the TM. One of three loudspeaker models was used in different experiments, ER-1 or ER-2 (both Etymotic Research Inc., USA), or Telex-1470 (Telex Communications, USA). The excitation signal was a multisine with linearly distributed frequencies. Measurements differed in frequency range (6, 8, and 10 kHz were used) and in number of FFT frequency bins (up to 512 points were used). The Transfer function was calculated from auto and cross spectra of input and output signal with 20 averages of the complex signals for each measurement. Measurements were quality checked by calculating the coherence between microphone and LDV signals. Only magnitude values with corresponding coherence greater than 0.8 were deemed valid, and the whole METF measurement was included in further analysis only if all points necessary for interpolation (see below, “Data format and preprocessing”) between 1000 and 4000 Hz were valid. The excitation signal was generated and measurements were recorded using data acquisition and control software programmed in LabView (National Instruments, USA) and corresponding hardware (PXI-4496, National Instruments, USA). TBs used were either fresh or freshly frozen. Data from this group will be denoted as “Dresden” and method “A”.

#### Medizinische Hochschule Hannover

The LDV used was either an HLV-1000 or an OFV-534/OFV-5000 (both Polytec GmbH, Germany). Instead of directly applying sound at the end of the bony part of the ear canal, the setup featured an ear funnel with a closed cavity cemented into the ear canal. This funnel and cavity was connected to the loudspeaker (DT48, Beyerdynamic, Germany), the microphone (ER-7C, Etymotic Research), and an observation window. The probe microphone was inserted to about 1–2 mm in front of the TM. The stimulation signal generation and acquisition was either performed by a 16 bit Polytec data acquisition system (PC-D and VIB-E-400, Polytec, Germany) or a 24 bit USB-4431 data acquisition system (National Instruments, USA) with a custom written software based on LabView (National Instruments, USA). A multisine signal of block size 1024 was presented at a sample rate of 25.6 kHz, which comprised equal signal magnitudes at 125 Hz, 250 Hz, 500 Hz, 1 kHz, 2 kHz, 3 kHz, 4 kHz, 6 kHz, 8 kHz, and 10 kHz. Outside of these frequencies, the multisine signal contained no excitation energy. This allowed for quality checking of the measurements by calculating the signal to noise ratio (SNR) directly from the measurement file: the measured magnitude value at the frequency points above was divided by the mean value of the six adjacent points. Because of the narrow-band excitation signal, these adjacent frequency points do not contain any useful signal and were therefore deemed a good approximation of the background noise. Measurement values with an SNR greater than 12 dB were considered to be valid. Data points were commonly averaged ≥ 30 times. The LDV’s angle of incidence $\Theta$ on the SFP was estimated during measurement and documented to allow for an angle correction of the data. The TBs used were freshly frozen. Data from this group will be denoted as “Hannover” and method “A”.

#### Fudan University Shanghai

The group used a CLV 2534-4 LDV with a VIB-E-220 acquisition unit and corresponding VibSoft-20 acquisition software (all Polytec GmbH, Germany) for measuring SFV. The microphone (ER-7C, Etymotic Research) was inserted into the auditory canal along with the earphone (ER-4PT, Etymotic Research) probe. The microphone probe was situated at about 1–2 mm in front of the TM. Excitation signals were single sine waves generated using a wave generator (Agilent 33210A 10 MHz) and an amplifier (B&K type 2718). LDV angle of incidence was not recorded but documented to be $\Theta \le 30^\circ$.

Velocity measurements were averaged 30 times and quality-checked using SNR for each frequency, with a threshold of SNR > 10 dB below 1 kHz and SNR > 20 dB for higher frequencies^15^. Data from this group will be denoted as “Shanghai” and method “A”.

#### Universitätsspital Zürich

The Zürich lab provided data in three distinct subgroups (see below). All TBs were prepared as per the general description above, but the outer ear canal was removed to about 2 mm from the tympanic annulus and replaced by an artificial ear canal of about 0.5 ml volume. This artificial ear canal also contained the earphone (ER-2, Etymotic Research) and probe microphone (ER-7C, Etymotic Research). The tip of the probe was set to a distance of about 3–5 mm from the center of the TM. The excitation signal used was either a multisine signal or multiple single sine signals. The TBs were treated with 0.1% Cialit (sodium-2-ethylmercurimer-capto-benzoxazol-5-carbonide) as a preservative agent.

The first subgroup was measured with a single point LDV as per the general description above Measurements from this subgroup were obtained using defrosted TBs only. The data is labeled as method “A”.

The other two subgroups of data both used multiple (50–100) measurement points on the SFP using a scanning LDV system (Polytec OFV-3001), but differed in processing of the data. The second subgroup calculated SFV as the complex mean value of the velocities measured. Measurements from this subgroup were obtained using both fresh and defrosted TBs. The data is labeled as method “B”.

The third group derived the piston-like component of the SFV from oval window LDV velocity scanning and X-ray microtomography (µCT). Upon fixating the reflective beads used for the SFV measurement in their respective positions, a 3D computer model of the TB was then rendered using µCT data. By combining SFV and µCT data, the 3D movement of the SFP and thus volume displacement was derived. For the sake of comparison, this derived data was then converted into an equivalent fundamental mode (piston-like) movement of the oval window (two higher modes corresponding to rocking motions were also calculated but not used in this study). Measurements from this subgroup were obtained using fresh TBs only. The data is labeled as method “C”. Its methods are explained in more detail in the related original studys^7,16^.

Data from this group will be denoted as “Zürich” with further distinction by measurement method as previously defined.

#### University of Colorado

Rather than at the SFP, in this group the LDV (either OFV-5000 and OFV-534 or HLV-1000 and CLV-700, all Polytec) was pointed at the stapes capitulum (head of the stapes). The resulting measurement is therefore stapes head velocity (SHV) instead of SFV. The angle of incidence was fixed at approximately $\Theta =45^\circ$. The reference microphone (either Bruel & Kjær 4182 or G.R.A.S 46BF) was inserted into the ear canal along with the loudspeaker (either Pyle PDWP5BK or Tucker Davis Technology), with the tip of the probe about 1 mm away from the TM. Excitation signals were generated with a custom made MATLAB program, and consisted of a series of single sine waves for multiple frequencies starting at 100 Hz and spaced logarithmically at ¼ octave intervals. Only freshly frozen TBs were used. The collected data from this group^17,18^ will be denoted as “Colorado”. Because the point of measurement is different, we label this data as method “D”.

**Table:** 

Laboratory	Method	TB state	Ref. Mic. position (mm)	Output signal	Angle correction	Angle	Total # of TBs
Dresden	A	Both	2–3	LDV SFV one-point	No	$\Theta <$ 30°	82
Hannover	A	Defrosted	1–2	LDV SFV one-point	Yes	$\Theta$ avg 42°	268
Shanghai	A	Defrosted	1–2	LDV SFV one-point	No	$\Theta <$ 30°	9
Zürich	A	Defrosted	3–5	LDV SFV one-point	No	$\Theta <$ 30°	42
Zürich	B	Both	3–5	LDV SFV scanning, complex mean value	No	40° $\le\Theta \le$ 50°	23
Zürich	C	Fresh	3–5	Piston-like component derived from LDV SFV scanning and µCT data	Yes	40° $\le\Theta \le$ 50°	36
Colorado	D	Defrosted	1	LDV SHV one-point	Yes	$\Theta \approx 45^\circ$	18

### Grouping and pre-processing of the data

#### Data format and preprocessing

The processing of the data was undertaken using the data analysis software OriginLab 2019, and the subsequent analysis was done in the programming language R 3.6.1^19^, using the IDE (integrated development environment) RStudio 1.2.5019. The raw data and the programming code containing the libraries and commands used are appended in the supplemental material.

The raw data was first checked for integrity. The different groups used different methods for data quality management as described above (e.g., based on SNR or coherence). Frequency resolution in data acquisition varied between measurements, from multisine signals (64–1024 frequency bins at sample rates of 12–25.6 kHz) to superposition of specific frequencies and single sine wave excitement. In addition, different frequency ranges were used. We therefore selected the following frequencies to represent the data: 125 Hz, 250 Hz, 500 Hz, 1 kHz, 2 kHz, 3 kHz, 4 kHz, 6 kHz. These are commonly used frequencies in audiological measurements, and all of the data includes measurements in this frequency range. All measurements were interpolated to these frequency points as necessary. We used linear interpolation here because any higher order interpolation would introduce bias towards one of the two interpolation points. Analysis on the METF magnitudes was performed in logarithmic scale.

For the analysis in R, the data is collected into data frames (see Fig. 1) where each row represents a single data point and the columns represent variables TB, frequency, METF (magnitude value), research group (Colorado, Dresden, Hannover, Shanghai and Zürich), measurement method (A = single point LDV SFV, B = multipoint LDV mean, C = volume displacement, D = single point LDV SHV), and angle correction (yes or no). The Hannover data and Zürich group C feature a quantified recording of $\Theta$, while other groups only ensured a rough adherence to a fixed angle as described above. In order for the data to be consistent, the uncorrected data is used in the main analysis below.

#### Detection of outliers

Even though all TBs measured were surgically inspected and METFs diligently acquired, measurement errors or undetected pathologies may occur. Such samples do not belong to the target population and must therefore be precluded from further analysis. A standard approach^20^ to detect outliers is to extend the first and third quartile ($Q1$ and $Q3$) by subtracting/adding a $k$-multiple of the interquartile range (IQR). Any data points outside this extended range between $Q1-k\cdot IQR$ and $Q3+k\cdot IQR$ are then considered outliers^21^. For normal distributions $k=1.5$ is most commonly used. Here we are using $k=1.75$ because the data does not have a completely ideal normal distribution (see “Overview and validation of the data”).

METFs with data points outside of these bounds were discarded in their entirety if the outliers were found in the range 500 Hz–4 kHz, and truncated at the more medial frequency if the outliers were detected only at the lower ($f<500 {\text{Hz}}$) or higher ($f>4 {\text{kHz}}$) end of the spectrum (or both, but not in between). We chose these bounds because from our own experience, the data in this middle range is usually most reliable and this frequency range is also the most important for most speech-related questions. The reason for completely discarding the whole METF is that a single outlier is potentially part of a wider peak, so it cannot be ruled out that neighboring frequency points may still be biased without being detected as outliers themselves. Note that quality checking, interpolation and outlier detection are performed sequentially in this order (see Fig. 1).

Using this method, we excluded a number of samples from each group (see Table 1). Note that due to the branched analysis (see Fig. 1), outlier detection was performed twice separately: first, outliers were removed in each data group, i.e., research groups sub-grouped by measurement method (left side of Fig. 1) to correctly make comparisons between the groups. Second, all uncorrected data sets pertaining to method “A” were combined into a large group, and outlier detection was performed on this combined set (right side of Fig. 1). Table 1 lists the exclusion by subgroup and for the combined group.

### Statistical analysis

#### Data comparison and evaluation of the lab effect

As described above, the data can be classified by two factors: research group and measurement method. We define methods based on documentation of experimental setup and procedure as described above. Any variability in a research group's data that is tied to that specific laboratory, but which cannot be attributed to known differences between the methodologies, will be called “lab effect”. The lab effect therefore includes any number of unknown causes.

The samples within groups are independent, and the groups are also independent. There is a dependence between frequency points *within each METF*, meaning that we have to assume that the magnitude at one frequency is influenced by the neighboring magnitudes in a non-negligible way. We have to assume heteroscedasticity of the data (different data sets have different variance). We therefore use multiple Welch^22,23^ t-tests with $\alpha =0.05$ to assess differences between groups at each frequency, because these are quite robust against non-normal distributions^24,25^. We are using the Holm method of sequentially rejective testing to avoid summation of type I errors (false positives)^26^ and denote the corresponding adjusted p value as ${p}_{adj}$. The local null hypotheses here are pairwise equality of the comparison groups, and the global null hypothesis is a frequency-wise equality of all comparison groups (once categorized by research group and once by measurement method).

The data is to be used in constructing a general reference range for the validation of METF measurements. This requires proof that any unspecified covariables have a negligible effect. It is therefore especially important to discern how the different parameters contribute to the overall variance. A method well suited to the task is to create a multivariate linear mixed model (LMM, as described by Winter^27^ or Harrison^28^) for these parameters, which are divided into “fixed effects” and “random effects”. In the present case, the parameter “frequency” as a within-groups factor was declared as a fixed effect variable, assuming that all METFs follow a similar frequency dependent pattern. The typical frequency response of the METF as known from literature suggests modeling as a 3rd order polynomial. However, we are also comparing higher order models to see whether accuracy can thus be improved. The parameters “measurement method” and “research group” as between-groups factors are considered random effect variables for the purpose of this evaluation. The inter-specimen variance also needs to be taken into account here and was introduced as random effect “TB”. Random effects are modeled as random intercepts. We use a crossed random effects model because for some levels of the factor “research group” there are multiple factor levels “measurement method”, therefore the use of a nested design as explained by Krzywinski^29^ is not appropriate. The LMM was implemented with the R package “LME4”^30^.

A common statistical measure for evaluating the polynomial fit is the coefficient of determination ${R}^{2}$ . It is computed here by the method of Nakagawa and Schielzeth^31^ and split into marginal ${R}^{2}$ (i.e. fixed-effects only) and conditional ${R}^{2}$ (fixed and random effects combined). The individual variance for the random effects obtained from the model-fit summary allows for distinguishing the various contributions to the model's overall variance. These different contributions can then be compared to gain insight into the relative variance of the parameters.

#### Data consolidation and calculation of reference confidence levels

The proposed reference range for individual TBs was calculated as a two-sided TI of the combined data set for method “A”. The $\left(p, 1-\alpha \right)$ TI is the statistical interval in which a specified proportion of $p\cdot 100\%$ of the population falls with a confidence level $\left(1-\alpha \right)\cdot 100\%$^11^. We set $\alpha =0.05$ because this is the most commonly used type I error rate and calculated the TI for $p\in \left[0.90, 0.95, 0.99\right]$. For better readability, we will use the shorthand $p\cdot 100\%$ TI for the $\left(p, 0.95\right)$ TI. The calculation of the TI was undertaken with the R package “Tolerance”^32^. In addition, we give the mean and 95% confidence interval of the mean for the combined data set.

## Results

### Overview and validation of the data

**Figure 2:**
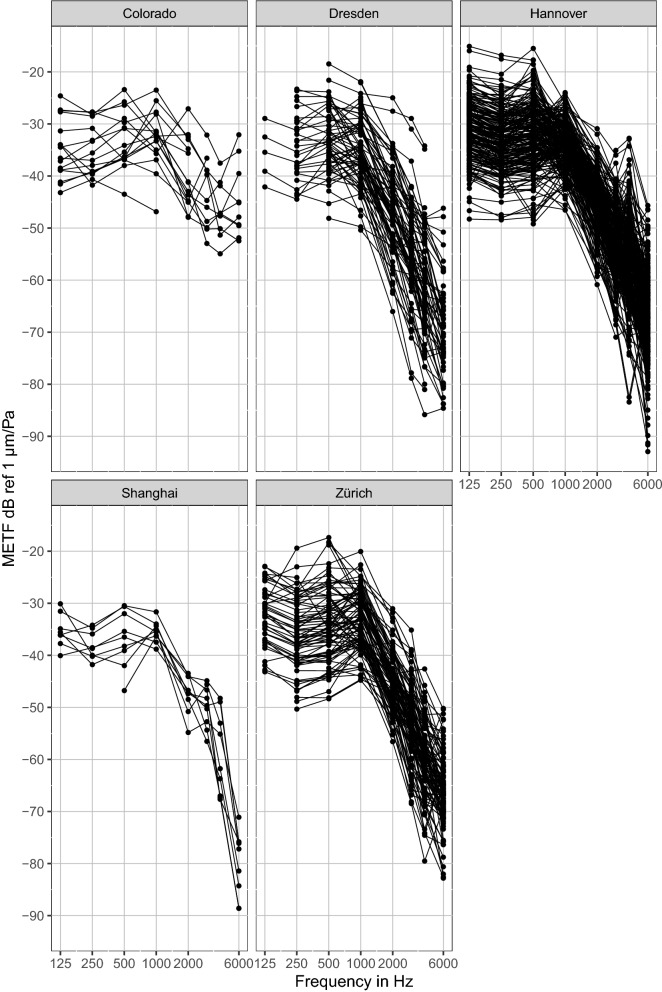
Collection of raw METF-data. METF data available for this study from all research groups and all measurement methods. The data has been quality checked and interpolated to audiological frequency points but no further preprocessing or selection has been applied at this step.

We will first look at the differences between groups (left side of Fig. 1). Plotting the interpolated raw data shows that it roughly follows the expected METF curve (Fig. 2).

**Figure 3:**
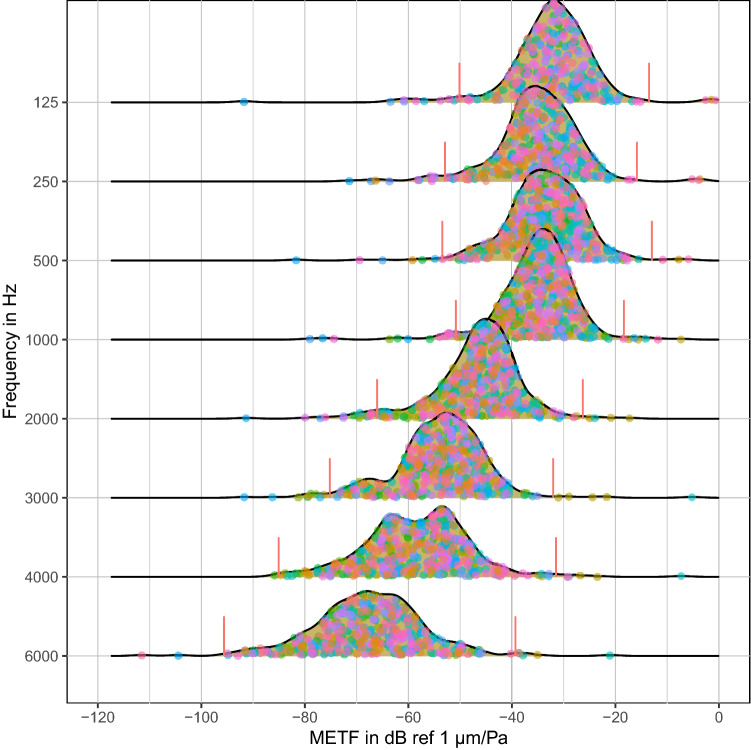
Ridge plot of method “A” METF data. The plot shows continuous probability density functions calculated with kernel density estimation. 1.75xIQR threshold boundaries for outlier detection are marked with red vertical borders. Similar plots for individual groups are included in the supplementary materials.

A closer look at the distribution density of the combined group for method "A" (Fig. 3) shows a slightly left-skewed distribution at lower frequencies up to about 1 kHz. Note that the data has been logarithmically transformed. Any data which is normally distributed in the log-normal transformed state are in fact left-skewed in their original state before transformation^23^. In the case of METF data the original data is left-skewed so much that it is visible even after transformation. At higher frequencies ($f\ge 4000 {\text{Hz}}$), kurtosis and outlier thresholds increase visibly ($1.75\cdot IQR$ values are marked), with clusters of non-outlier data points at various magnitudes. Skewness and kurtosis are < 2 for all distributions, all values for these are given in the supplements. By the rules defined above (“Detection of outliers”), there are some outliers at all frequencies.

### Differences between data sets

#### Lab effect and research group effects

**Figure 4:**
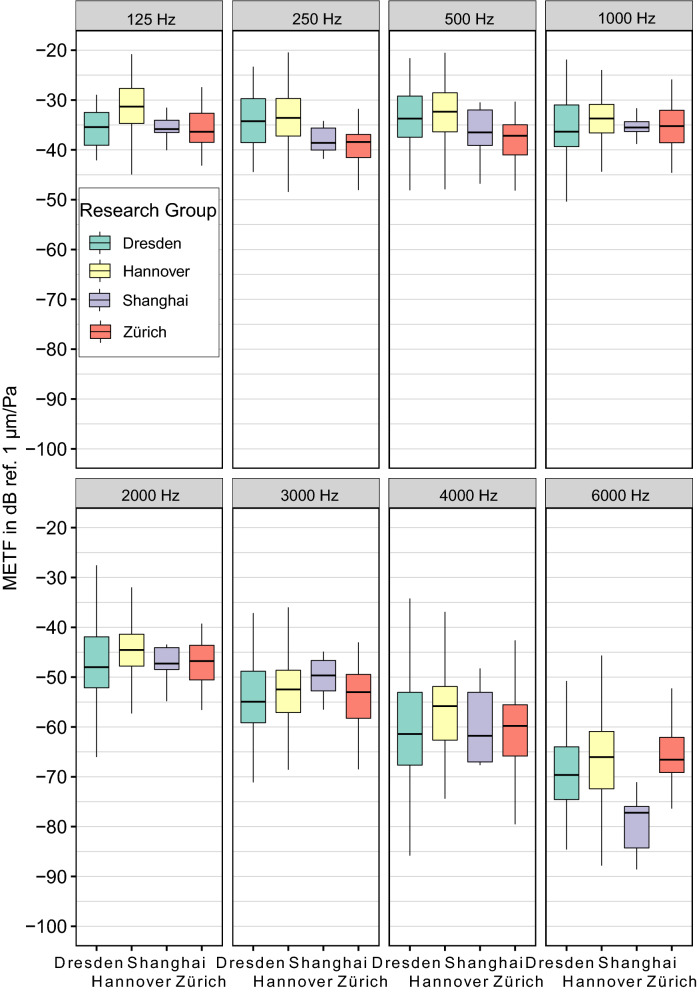
Comparison of METF data from different research groups by frequency. All groups use single point LDV measurements on the stapes footplate as output signal (method A). Median Magnitudes and quartiles without outliers are depicted. Complete results of the corresponding Welch t-tests are listed in supplements.

Welch t-test analysis of the data sets for method "A" shows that in the middle band (1–3 kHz), there are no significant differences (for an adjusted alpha = 0.05) between research groups. However, there are some significant mean differences between some research groups at the low and high end of the spectrum (see Fig. 4). At $f\le 500$ Hz these differences are 6 dB or less. At 125 Hz, the Hannover mean deviates from all other groups by about 4–5 dB. The other means are within 1 dB of each other. The differences between the Hannover mean and the other means are significant in the cases of Shanghai (${p}_{adj}=0.035$) and Zürich (${p}_{adj}=0.01$), but not significant in the case of Dresden. It is important to note that at this frequency, there are 225 data points in the Hannover data set—many more than in the other groups (Dresden: 7, Shanghai: 9, and Zürich: 14). At higher frequencies, the Shanghai data drops off faster than the others, with a significant difference of 11 dB compared to Dresden (${p}_{adj}=2\times {10}^{-3}$), 13 dB compared to Hannover (${p}_{adj}=6\times {10}^{-4}$), and 15 dB compared to Zürich (${p}_{adj}=1.97\times {10}^{-4}$) at 6 kHz. Please refer to the supplemental material for complete t-test results. These differences can be attributed partly to known differences in the methods used.

#### Measurement method

**Figure 5:**
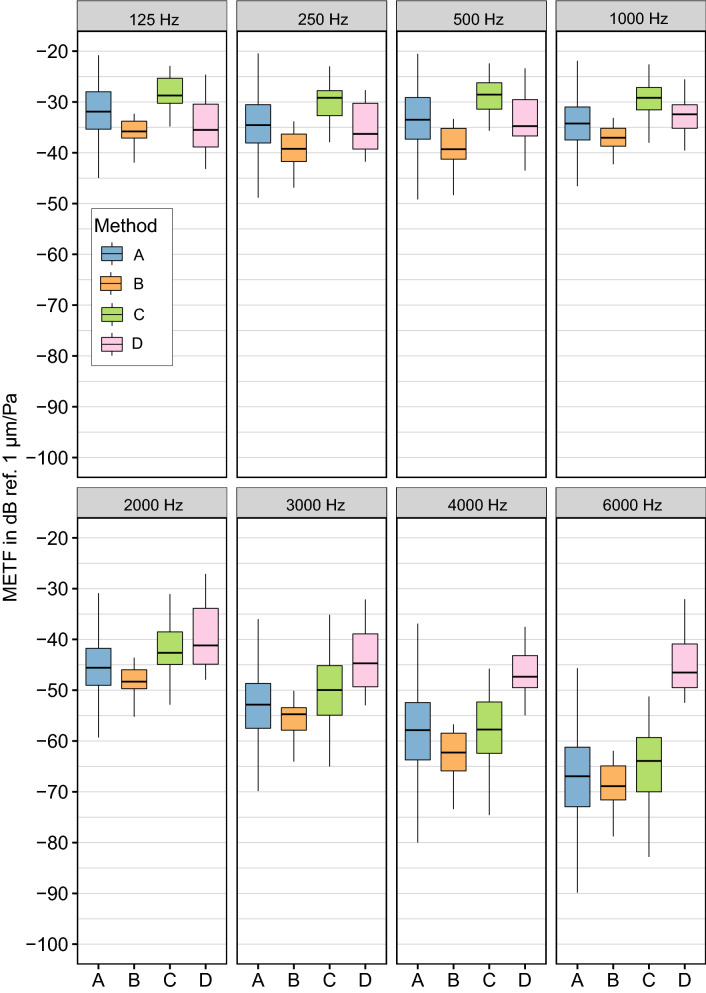
Comparison of measurement methods by frequency. Median magnitudes and quartiles without outliers are depicted. (A) single point LDV on footplate, (B) scanning LDV mean, (C) volume displacement calculated from scanning LDV data, (D) single point LDV on stapes head. Data for methods (A), (B) and (C) is from the same lab (Zürich), the data for method (D) is from another lab (Colorado). The data of method (A) from other research groups was excluded here in order to focus on differences between methods and not research groups. The results of the corresponding Welch t-tests are listed in supplements.

As described above (see “Laboratory specifics”) even though the data from the Zürich group was all acquired as SFV, it includes three fundamentally different methods of measuring or deriving single point scalar data. The Colorado data was acquired using a different point of measurement and is therefore classified as a fourth method. This allows for a comparison between the different methods regarding the outcome. It must be noted that the data also differs in TB state and angle correction (see Table 1). We compare the combined data sets for each of the four methods by using Welch t-test analysis. The results show an ascending order of magnitudes (“B”, “A”, then “C”) for the first three methods at all frequencies (see Fig. 5). These differences of up to 10 dB are significant for the most of the frequency range.

Up to 1 kHz, Method “D” results are similar to those for method “A” but do not show the typical drop with increasing frequency (also compare Fig. 2). This results in gradually greater differences. At 6 kHz, these have grown to 21 dB (${p}_{adj}=1.11\times {10}^{-6}$) compared to A, 24 dB (${p}_{adj} = 2.4\times {10}^{-7}$) compared to B, and 20 dB (${p}_{adj}=6.7\times {10}^{-7}$) compared to C. As before, t-test results are given in the supplements.

#### Angle correction

Data for the Hannover group was supplied both with and without angle correction. This allows for a direct calculation of the influence of this parameter on the METF data. $\Theta$ varies from 0° to 60°, with a mean of 42°^33^ which corresponds to a signal change of 2.6 dB. Assuming that the necessity for a less-than-ideal angle is universal, this is a good approximation of the mean error when angle correction is not applied. Note that the maximum error in the groups that limited maximum angle to 30° and 45° are roughly 1.25 dB and 3 dB, respectively.

### Linear mixed model analysis

The third order polynomial fit describes the mean of the data very well. The LMM analysis gives a marginal ${R}^{2}$ of 71% and a conditional ${R}^{2}$ of 86%. Higher order polynomial models do not increase accuracy meaningfully (see Supplements). The model shows 17.6 dB variance from “TB” and 11.5 dB from “measurement method”. The variance from “research group” is only 1.98 dB. The corresponding standard deviations are 4.2 dB for “TB”, 3.4 dB for “measurement method” and 1.4 dB for “research group”. The corresponding R script and results for the parameters are given in detail in the supplements.

### Mean and statistical ranges

**Figure 6:**
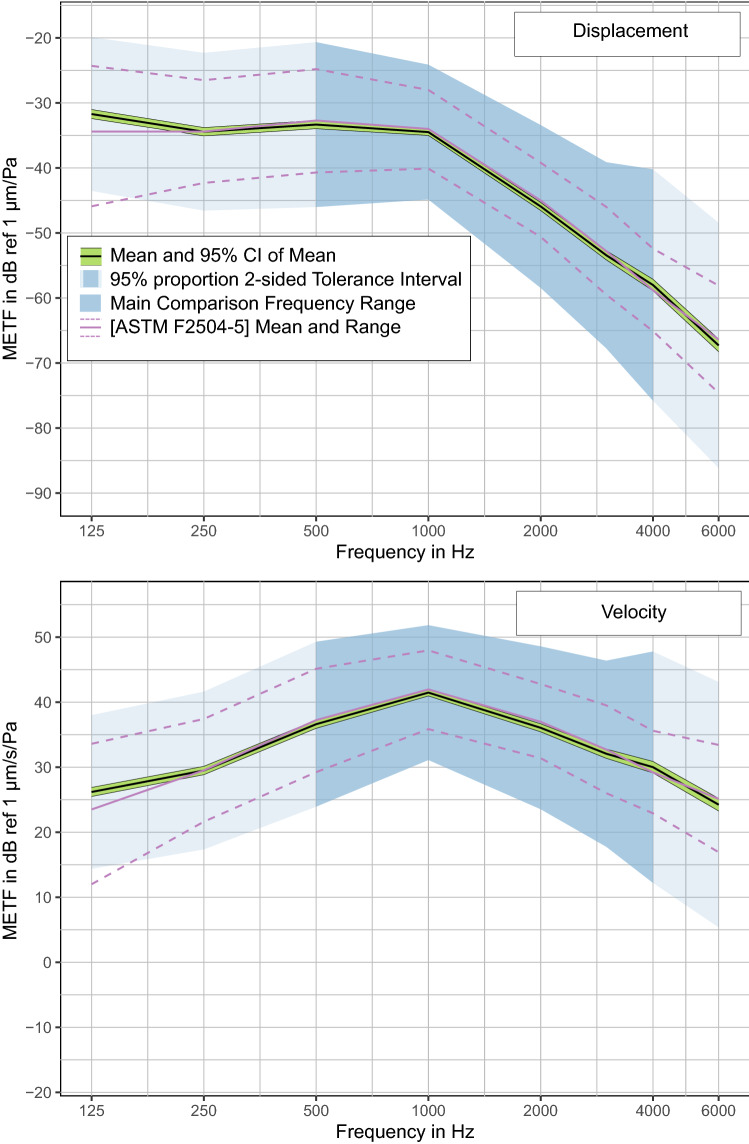
Proposed reference for METF measurements. The reference range is calculated as the 2-sided 95% tolerance interval. Upper: stapes footplate displacement in dB ref µm/Pa. Lower: stapes footplate velocity in dB ref µm/s/Pa. Inferential statistics from 366 METF measurements based on measurement method "A" (single point LDV on stapes footplate) from 4 research groups (Dresden, Hannover, Shanghai, Zürich). Literature (ASTM) range is included as comparison.

Selecting all interpolated data sets from all groups for method "A" gives a total number of 401 TBs, with a total of 2993 number of data points over 8 frequency points. This combined data set was then checked for outliers using the detection method described above (right side of Fig. 1), which leaves 366 TBs and 2321 total data points. The results for the mean and 95% CI of the mean and the 95% TI are given in Table 2 and Fig. 6 (also see supplementary material for a spreadsheet version that also includes the 90% TI and 99% TI). All values were calculated as stapes footplate displacement and velocity. The corresponding data sets are given in the supplements.

**Table 2:** Numerical values of METF tolerance interval ranges.

Frequency in Hz	METF in dB ref. 1 µm/Pa			METF 2-sided tolerance intervals (α = 0.95) in dB ref. 1 µm/Pa	
	Mean	95% confidence interval of mean		95% population proportion	
		Lower boundary	Upper boundary	Lower boundary	Upper boundary
**SFD—stapes footplate displacement**					
125	− 31.6	− 32.2	− 30.9	− 43.2	− 19.9
250	− 34.2	− 34.8	− 33.6	− 46.1	− 22.3
500	− 33.2	− 33.8	− 32.5	− 45.8	− 20.5
1000	− 34.4	− 34.9	− 33.8	− 44.7	− 24.0
2000	− 45.7	− 46.3	− 45.0	− 58.0	− 33.3
3000	− 53.2	− 54.0	− 52.5	− 68.1	− 38.4
4000	− 57.8	− 58.7	− 56.9	− 76.0	− 39.6
6000	− 67.0	− 68.0	− 66.1	− 85.5	− 48.7

Frequency in Hz	METF in dB ref. 1 µm/s/Pa			METF 2-sided tolerance intervals (α = 0.95) in dB ref. 1 µm/s/Pa	
	Mean	95% confidence interval of mean		95% population proportion	
		Lower boundary	Upper boundary	Lower boundary	Upper boundary
**SFV—stapes footplate velocity**					
125	26.3	25.6	27.0	14.7	38.0
250	29.7	29.1	30.3	17.8	41.6
500	36.8	36.1	37.4	24.1	49.4
1000	41.6	41.1	42.1	31.3	52.0
2000	36.3	35.7	36.9	24.0	48.7
3000	32.3	31.5	33.0	17.4	47.1
4000	30.2	29.3	31.1	12.0	48.4
6000	24.4	23.5	25.4	6.0	42.9

All values are given as displacement (upper panel) and velocity (lower panel). Please refer to the supplementary material for a comma-separated spreadsheet version of this table.

## Discussion

### Preprocessing and validation

The data sets at each frequency point have a slightly left-skewed (negatively skewed) distribution (see Fig. 3). This is to be expected, because the middle ear acts as an impedance matching mechanism, but does not comprise any active amplification. There is therefore a natural upper limit to the sound transfer characteristics, while the (theoretical) lower limit due to attenuating effects such as ossification or ligament stiffening is zero transfer^34^. The data can be considered normally distributed for purposes of the following analysis because both skewness and kurtosis are less than 2 and the sample size is large^25,35,36^.

Even though all data has been diligently acquired, measurement errors cannot be ruled out. In addition, there is no way of directly identifying false data, because errors present in the signals may interfere with (“hide”) one another, so that the resulting METF appears normal. However, there are some clusters of outliers which are possibly measurement errors. Using 1.5 IQR as exclusion criteria would eliminate these, but due to the negative skewness of the distribution, also a large part of the total (53 of 401). The choice of 1.75 IQR is more lenient and excludes 35 of the 401 samples, therefore it seems to offer a better balance between excluding obvious errors and including TBs which are untypical but still normal.

Note that we are trying to establish a range of sound transfer in normal ears. It is possible that there are individuals in which sound conduction is very different from the rest of the population, but who still have normal hearing. However, we cannot confirm this because there is no assignment of hearing tests to temporal bones. Therefore, to be on the safe side, we have to assume that outliers are the result of undetected pathologies or measurement errors.

### Data comparison

From the Welch T-tests between groups belonging to method "A", we can see there is a lab effect (as defined in “Statistical Analysis”, see Fig. 4), but it is only statistically significant at the edges of the frequency range.

The t-tests between all methods further show that there are significant differences between the methods (see Fig. 5), although not at all frequencies. This can be interpreted as a first indication that the different methods are systemically dissimilar, but that for the most part, all data labeled as method "A" is comparable.

The LMM analysis gives a deeper understanding of these notions:

1. The variance is strongly influenced by the different methods. This confirms the classification of methods "B", "C" and "D" as distinct measurement methods.
2. The lab effect is small compared to the TB variance (2 dB vs 18 dB). It follows that the data for method “A” from all research groups may be combined into a common data set from which to derive the desired reference range. By the same logic, this range is then also applicable to new data from any other lab, provided the measurement methodology is documented in sufficient detail and is consistent with method "A" described here.

A recently published modal analysis of METF measurements shows a rather large standard deviation for the frequency distribution of the natural frequencies of the middle ear^37^, which contributes to the large interspecimen variance. A parametric evaluation of the METF data as published by Gladiné^38^ may therefore be an interesting subject of further research.

The reasons for the detected lab effect are confounding variables that are not covered by the level of detail of the experiment definition. In other words, the lab effect collects all unexplained differences between the setup and procedure for the different research groups. Some variables that may be worth investigating further are TB state (fresh or frozen), sex, and age of the donor at time of death. It has been shown that input impedances of the middle ear are unaffected even by prolonged freezing^39^, but sound transmission may be affected by other structural changes due to freezing/thawing^40–43^. It is as of yet unclear whether or how exactly age at death correlates with the METFs measured in TBs. Simulations suggest that there may be symptomatic changes^44^, and there are some studies that have examined the age dependence of sound transfer characteristics^9,45,46^. It has been shown that sex influences both the anatomy^47^ and middle ear function^48^. However, in all of these cases the effect on the results of this study is likely small, as evidenced by the small overall lab effect. Note that any change leading to pathological behavior (such as perilymph leakage or hypermobility due to damaged ligaments) disqualifies TBs in the preselection process^8^. TB state, sex and age impact on the METF may be the focus of future studies, possibly leading to separate validation ranges for fresh and defrosted TBs, for different sexes, or for different age ranges.

METF measurements in TBs are insensitive to varying microphone positioning up to 4 mm from the umbo^14^ because at the observed frequency range, the wavelengths of the sound waves are far larger than this. An impact on the results is therefore unlikely, because only the Zürich group potentially contained data measured at a distance of up to 5 mm. It may be advisable to narrow this down further in future studies if possible. The point of measurement on the SFP may account for a part of the variability, but this cannot be quantified on the basis of the data. A recent study by Cheng et al. indicates that nonlinear transmission effects may occur at sound pressure levels as low as 100 dB SPL, with expansive behavior dominant in the frequency range below 2 kHz and compressive behavior towards higher frequencies^49^. The METF measurements used here use excitement levels of about 90–100 dB SPL. Even though occasionally higher SPL may be used to acquire a better signal, the effects are likely very small at this point.

It can be expected that the lab effect would be smaller with a stricter definition of the experimental design for obtaining METF measurements, such as including an age range for the specimens, more exact measurements of microphone position, point of measurement on the SFP, adhering to an identical preparation method and application of the loudspeaker etc. However, it is much more practical to provide a validation range for the less restrictive setup definition as this is immediately applicable for a larger number of research teams. Having proven that the lab effect is relatively small despite the variability between setups also justifies this approach.

It must be noted that at the 125 Hz sampling point, the Hannover data dominates the calculations due to the higher number of valid data points. At 6 kHz, the data from Shanghai is significantly lower than the rest. Therefore, the reference intervals are somewhat less reliable at the low and high end of the range, which is addressed in Fig. 6 by highlighting the more reliable frequencies as "main comparison frequency range". The resulting difference is small; however, it should be kept in mind. This effect can be mitigated as more data from the other labs becomes available.

### Reference intervals

Compared to the ASTM—where the reference range is calculated as a CI of the mean—the CI of the mean in the present study is a much tighter fit around the mean (less than 1 dB on each side, see Fig. 6). This is to be expected due to the large number of samples. It must be noted once more that the CI given in the ASTM is calculated for the mean of other studies' means, whereas here, both the mean and its CI are calculated from individual METFs. We are therefore choosing the associated TI as the proposed reference range, which spans an interval of about 20 dB. The TI is about 5–6 dB larger in both directions than the CI used in the ASTM standard^8^ which is shown in red dashed lines as a comparison. This is approximately in line with the "revised rules" of Rosowski^9^, which increases the range by 20% and states that deviations from the ASTM range by up to ± 6 dB may be acceptable. In contrast to this rule, which was inferred from personal experience, the TI range and mean presented here provide an objective basis. As has been pointed out^10^, the CI (on which the ASTM standard is based) is inversely proportional to the square root of $n$, and so a range constructed from larger $n$ would reject more individual data points. The ($p,1-\alpha$)-TI is calculated differently (see “Data consolidation and calculation of reference confidence levels”) and does not have this limitation.

We propose the TI ranges to be used as indicators for normal sound transmission in individual TBs if measurements from these are to be included in a statistical analysis. Such a proposed reference range should, on the one hand, reliably identify pathological TBs and false measurements. On the other hand, it should include most, if not all, normal METFs. The TI range presented here can be considered reliable in this aspect due to the large database from independent laboratories used in constructing it.

However, there is an important aspect to keep in mind when applying it as a reference. The large natural variability between samples as evident from the LMM results means that some TBs—though valid, non-pathological samples—transfer sound much better or much worse than others. In a smaller study, a single TB with an exceptional METF can have a big impact on the statistical results. This could lessen the meaningfulness of these results, even if the measurement itself is valid.

**Figure 7:**
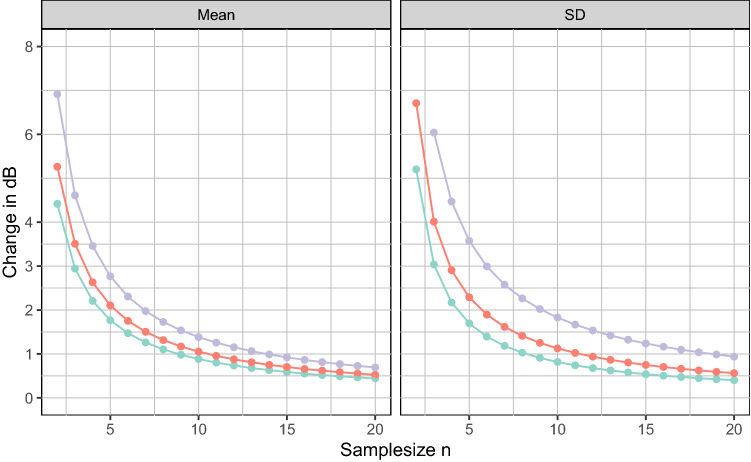
Impact of isolated extreme data in small studies. In a hypothetical future study that includes $\mathrm{n}$ samples, a single measurement at the edges of our proposed reference range (see Fig. 6) will affect the mean of the study (left) and the standard deviation (middle) as shown. We are considering the 90%, 95%, and 99% tolerance interval calculated as described in the text. Note that this illustrates a hypothetical experiment as detailed in the text.

Consider a hypothetical new study that includes $n$ TBs. All of the new study's METFs are completely within the reference range, however, one METF measurement lies right at the edge of the range. Assuming that the variance of the new study's data is similar to the reference, we can calculate the influence of a single METF measurement at the edge of each of the possible ranges of our study (90%, 95%, and 99% TI) on the statistical results (mean and standard deviation) for various $n$ (see Fig. 7). The full derivation for this calculation is given in the supplements.

There are two main observations here: first, as can be expected, the impact of single extreme measurements on the study as a whole rises steeply for smaller sample sizes (n < 10). This is important to note for any future study. Second, the differences in outcome between the chosen 95% TI and the other TIs are relatively small. This means that in practice, there would be little difference in using a proportion of 90% or 99% instead. We therefore chose the 95% TI as the proposed reference range. While we realize that this choice is arbitrary, we feel that it is a good compromise between ruling *in* as many normal METFs, and ruling *out* as many pathological METFs as possible. Since the underlying reference data has been carefully selected and preprocessed, this accurately represents the population, excluding only 2.5% of all METFs at the high and low end, respectively.

Due to the large number of available data sets, the 95% confidence interval of the mean is very narrow, implying that the study mean is highly likely to accurately represent the mean of the population as a whole. This allows one to compare a new study’s mean to the reference mean. Note that it is unlikely that the mean of a new study using a smaller sample size would fall within this range. However, comparing the study mean with the reference mean can give insight on how well the new study population matches the whole population. This comparison should take into account a possible lab effect, which by the reference data can be expected not to exceed 6 dB. A larger difference between the means might be worth investigating.

### Suggested use of the reference data

We have derived statistical ranges for METF magnitudes from a population of TBs with normal, non-pathological sound transfer characteristics. These intervals are intended as a reference for future studies in a variety of contexts (for example, output of AMEI (Active Middle Ear Implants), reconstructive surgery or basic research) using similar methods. They allow validation of individual TBs and assessment of the sample group. For such use, we suggest the following guidelines.

1. Measure all METFs in a way similar to the procedure outlined above.
  In short, use a single point LDV near the center of the SFP to acquire the output signal and a probe microphone in front of the TM to acquire the input signal. Ensure that the LDV angle as measured from the normal on the SFP is as small as possible and note this angle along with the data.
2. Preprocess your data.
  Correct for the LDV angle if possible. Check the quality of the data (e.g. using coherence or SNR). As long as data quality is high in the frequency band of 500–4000 Hz, low quality data points at the high or low end of the spectrum (lower than 500 Hz or higher than 4000 Hz) may be truncated depending on the questions at hand.
3. Compare each METF in your study to the reference range. If an METF exceeds the range in one or more places, take note.
  Bear in mind that the ranges are only a tool to alert the researcher to an atypical measurement. It is up to you to determine whether this is due to a hitherto undetected pathology, a measurement error, or other causes. In some cases, the measurements from this TB may still be used, but this should be justified by the purpose of the study and discussed. In case of doubt, we suggest excluding the data set from the group statistics. Note that narrow band resonances/antiresonances may result in peaks/notches that exceed the range for an otherwise normal METF; these may be ignored but it may be advisable not to draw any conclusions from the data immediately at or around the resonance. If possible, use excitation signals with high frequency resolution (such as multisine signals) to better differentiate narrow band resonances from wider deviations.
4. Be aware that single measurements towards the (upper or lower) edges of the range may have a large impact on your analysis.
  As a rule of thumb, we propose that studies using statistical analysis use $n\ge 5$ and preferably $n\ge 10$ valid measurements, depending on the targets of the study.
5. Use your own judgment for case studies or special problems.
  The reference is intended for use in studies with a statistical analysis (i.e., using multiple TBs). If the measurements are to be examined as individual cases only, other criteria must be used to assess the validity of the measurements. The same applies if the target of your study is to examine middle ears with pathological aberration that explains the deviation from the range.
6. If an METF deviates only at the high or low end of the frequency range, it may be truncated instead of discarded.
  This greatly depends on the target of the study, but any deviation with $f>4 {\text{kHz}}$ or $f<500 {\text{Hz}}$ may be truncated accordingly if the aim of your experiment allows it (such as speech-related questions).
7. Compare the mean of your study to the reference mean.
  This can give an estimate of how well your study represents the whole population on average. Note that for small studies, it is highly unlikely that your mean falls inside the 95% CI of the reference mean. As a rule of thumb, if the mean of your study deviates from the reference mean by more than 6 dB, the possible implications thereof should be addressed in discussions.

The reference range and mean presented here are calculated from 362 METF measurements provided by 4 research groups. Its primary use is for such research where only a small number of measurements must be validated. In cases where a large number of data sets from the same experimental setup is available, it may be advisable to use the methods provided to calculate a reference range for this specific setup.

The original data, as well as the calculation scripts used, are attached and summarized in the supplemental material so that it may be compared, reproduced, or updated with future data. The statistical methods used can also be adapted for use with different measurement methods, or indeed for use in other experiments where transfer functions are subject to large anatomical variations.

## Supplementary Information

Supplementary Information 1.

Supplementary Information 2.

Supplementary Information 3.

Supplementary Information 4.

Supplementary Information 5.

Supplementary Information 6.

## Data Availability

All data generated or analyzed during this study, including the data sets from all contributing groups as well as the R scripts used are included in this published article and its supplementary information files.

## Abbreviations

AMEI: Active middle ear implant
ASTM: American Society for Testing and Materials
CI: Confidence interval
dBSPL: Decibel sound pressure level
dB SPLeq.: DB SPL equivalent
FFT: Fast Fourier transform
IQR: Interquartile range
LDV: Laser Doppler vibrometer
METF: Middle ear transfer function
OC: Ossicular chain
SFP: Stapes footplate
SFV: Stapes footplate velocity
SHV: Stapes head velocity
SNR: Signal to noise ratio
TB: Temporal bone specimen
TI: Tolerance interval
TM: Tympanic membrane
$\Theta$: LDV angle of incidence relative to normal of the SFP
