# Supplementary material for: Methods and reference data for middle ear transfer functions
Source: Sci Rep. 2022 Oct 14;12:17241. doi: 10.1038/s41598-022-21245-w (PMC9568555; doi:10.1038/s41598-022-21245-w)
Supplement: Supplementary file 3 — Supplementary Information 3. [file 41598_2022_21245_MOESM3_ESM.docx]

# Supplemental Materials

## Statistical analysis – R scripts and input raw data

Corresponding section in main article: 3.3 Grouping and pre-processing of the data

Corresponding figures in main article: All graphs.

Raw data for all calculations presented in the main article are available for download as a compressed folder “METF_Raw_Data.zip”. The statistical analysis uses scripts in the programming language R. The compressed folder "R_scripts.zip" contains all of these scripts. Together, the above may allow for a more detailed comprehension of the calculation in this study, or it may be used for adding additional METF data sets for comparison.

The R scripts are partitioned modularly and should be applied as follows (see Supplementary table S1).

1. Description of R scripts for statistical analysis

| **Step** | **Scripts** | **Notes** |
| --- | --- | --- |
| **1** | “001_Package_Load.R” | Clears the R dataspace and loads all packages necessary for the calculations. |
| **2** | “002_Input_Data…” to “008_Input_Data...” | Reads the raw data files into R and builds the base for an R data frame for the subsequent analysis. |
| **3** | “009_Collected_Input_Files_Environment.RData” | If the original database will be used, **step 2** can be skipped by directly loading the environment database into R. |
| **4** | “010_Evaluation_of_Data_Set_Differences.R”  “011_Calculation_Of_Reference_Ranges” | calculate the accordingly analysis as depicted in study outline schemata figure 1 of the study. |

Additional commentary is contained in the scripts.

## Lab effect and differences between datasets

Corresponding section in main article: 4.2 Differences between data sets

Corresponding figures in main article: Figure 4 and Figure 5

The T-Test-Results are appended as comma-separated spreadsheets in “T_Test_Results.zip”.

## Linear mixed model analysis – detailed results

Corresponding section in main article: 4.3 Linear mixed model analysis

1. Result of linear mixed model analysis

|  | **METF** | | |
| --- | --- | --- | --- |
| ***Predictors*** | ***Estimates*** | ***CI*** | ***p*** |
| (Intercept) | -44.22 | -48.01 – -40.44 | <0.001 |
| Frequency [1^st^ degree] | -680.96 | -691.70 – -670.22 | <0.001 |
| Frequency [2^nd^ degree] | 64.93 | 54.15 – 75.70 | <0.001 |
| Frequency [3^rd^ degree] | 60.37 | 49.66 – 71.08 | <0.001 |
| **Random Effects** | | | |
| $\sigma^{2}$ | 29.57 | | |
| $\tau_{00 TB}$ | 17.55 | | |
| $\tau_{00 research group}$ | 1.98 | | |
| $\tau_{00 measurement method}$ | 12.19 | | |
| $ICC$ | 0.52 | | |
| $N_{TB}$ | 432 | | |
| $N_{research group}$ | 5 | | |
| $N_{measurement method}$ | 4 | | |
| Observations | 3190 | | |
| marginal R^2^ / conditional R^2^ | 0.707 / 0.859 | | |

## Reference range – tolerance intervals

Corresponding section in main article: 4.4 Mean and statistical ranges

Corresponding figure in main article: Figure 6

Supplementary table S3 is an extended version of the corresponding Table 3 in the main article, which includes the 90% and 99% tolerance interval. A comma-separated spreadsheet of these results is available for download as “Tolerance_Intervals.zip”. The graphs of the tolerance intervals are also available as interactive HTML-files in “Tolerance_Intervals_interactive.zip”.

1. Descriptive statistics for reference data

| **SFD – Stapes Footplate Displacement** | | | | | | | | | | | | |
| --- | --- | --- | --- | --- | --- | --- | --- | --- | --- | --- | --- | --- |
| Frequency in Hz | METF in dB **ref. 1 µm/Pa** | | | METF 2-sided Tolerance Intervals (on a significance level of α=0.95)  in dB **ref. 1 µm/Pa** | | | | | | | | |
|  | Mean | 95% Confidence Interval of Mean | | 90% population proportion | | | | 95% population proportion | | | 99% population proportion | |
|  |  |  |  |  |  |  |  |  |  |  |  |  |
|  |  | lower  boundary | upper boundary | lower  boundary | upper boundary | | | lower boundary | | upper boundary | lower boundary | upper boundary |
| 125 | -31.7 | -32.4 | -31.0 | -41.5 | -21.8 | | | -43.4 | | -19.9 | -47.1 | -16.2 |
| 250 | -34.4 | -35.0 | -33.8 | -44.5 | -24.2 | | | -46.5 | | -22.3 | -50.3 | -18.5 |
| 500 | -33.3 | -33.9 | -32.7 | -43.9 | -22.6 | | | -45.9 | | -20.6 | -50.0 | -16.7 |
| 1000 | -34.4 | -34.9 | -34.0 | -43.2 | -25.6 | | | -44.8 | | -24.0 | -48.2 | -20.7 |
| 2000 | -45.9 | -46.5 | -45.3 | -56.4 | -35.3 | | | -58.5 | | -33.3 | -62.4 | -29.3 |
| 3000 | -53.4 | -54.1 | -52.7 | -65.4 | -41.4 | | | -67.7 | | -39.1 | -72.2 | -34.7 |
| 4000 | -58.0 | -58.9 | -57.1 | -72.9 | -43.0 | | | -75.8 | | -40.2 | -81.4 | -34.6 |
| 6000 | -67.3 | -68.3 | -66.3 | -83.1 | -51.5 | | | -86.2 | | -48.4 | -92.1 | -42.5 |
| **SFV – Stapes Footplate Velocity** | | | | | | | | | | | | |
| Frequency in Hz | METF in dB **ref. 1 µm/s/Pa** | | | METF 2-sided Tolerance Intervals (on a significance level of α=0.95)  in dB **ref. 1 µm/s/Pa** | | | | | | | | |
|  | Mean | 95% Confidence Interval of Mean | | 90% population proportion | | | 95% population proportion | | | | 99% population proportion | |
|  |  |  |  |  |  |  |  |  |  |  |  |  |
|  |  | lower boundary | upper boundary | lower boundary | | upper boundary | lower boundary | | upper boundary | | lower boundary | upper boundary |
| 125 | 26.2 | 25.5 | 26.9 | 16.4 | | 36.1 | 14.5 | | 38.0 | | 10.8 | 41.7 |
| 250 | 29.5 | 28.9 | 30.2 | 19.4 | | 39.7 | 17.5 | | 41.6 | | 13.7 | 45.4 |
| 500 | 36.7 | 36.0 | 37.3 | 26.0 | | 47.3 | 24.0 | | 49.3 | | 20.0 | 53.3 |
| 1000 | 41.5 | 41.0 | 42.0 | 32.8 | | 50.3 | 31.1 | | 52.0 | | 27.8 | 55.3 |
| 2000 | 36.1 | 35.5 | 36.7 | 25.5 | | 46.7 | 23.5 | | 48.7 | | 19.6 | 52.6 |
| 3000 | 32.1 | 31.4 | 32.8 | 20.1 | | 44.1 | 17.8 | | 46.4 | | 13.3 | 50.9 |
| 4000 | 30.0 | 29.2 | 30.9 | 15.1 | | 45.0 | 12.2 | | 47.8 | | 6.6 | 53.4 |
| 6000 | 24.2 | 23.3 | 25.2 | 8.4 | | 40.1 | 5.4 | | 43.1 | | -0.6 | 49.0 |

## Impact of extreme data - derivation of calculations

Corresponding section in main article: 5.3 Reference intervals

Corresponding figure in main article: Figure 7

From the main text: " Consider a hypothetical new study that includes $n$ TBs. All of the new study's METFs are completely within the reference range, however, one METF measurement lies right at the edge of the range."

The arithmetic mean $\bar{X}$ and empirical standard deviation $SD$ are given by

$$\bar{X}=\frac{1}{n}\sum_{i=1}^{n} x_{i}$$

$$SD=\left( \frac{1}{n-1}\sum_{i=1}^{n} \left( x_{i}-\bar{X} \right)^{2} \right)^{\frac{1}{2}}$$

In order to assess the impact $\Delta\bar{X}$ of this single extreme value $\tilde{x}_{n}$ on the mean, we will first assume that the $n$^th^ value in the above sum does not contribute to $\bar{X}$ at all, i.e.,

$$\frac{1}{n}\sum_{i=1}^{n-1} x_{i}=\frac{1}{n}\sum_{i=1}^{n} x_{i}$$

We then substitute $\tilde{x}_{n}$ for $x_{n}$ and subtract the original mean from the resulting new mean $\tilde{X}$:

$$\Delta\bar{X}=\tilde{X}-\bar{X}=\frac{1}{n}\sum_{j=1}^{n-1} x_{j}+\frac{1}{n}\tilde{x}_{n}-\frac{1}{n}\sum_{i=1}^{n} x_{i}$$

The first and last terms above are both identical to $\bar{X}$. Thus

$$\Delta\bar{X}=\frac{1}{n}\tilde{x}_{n}$$

with $\tilde{x}_{n}$ being set equal to the outermost value of the tolerance interval reference range (Table 4). The impact on $SD$ is derived similarly using the definition of $SD=\sqrt{S^{2}}$, where $S^{2}$ is the empirical variance. Again assuming a case where the $n$^th^ term does not contribute, we can write

$$\Delta SD=\tilde{SD}-SD=\left( \underset{S^{2}}{\underbrace{\frac{1}{n-1}\sum_{j=1}^{n-1} \left( x_{j}-\bar{X} \right)^{2}}}+\underset{\beta}{\underbrace{\frac{1}{n-1}\left( \tilde{x}_{n}-\bar{X} \right)^{2}}} \right)^{\frac{1}{2}}{-\left( \underset{S^{2}}{\underbrace{\frac{1}{n-1}\sum_{i=1}^{n} \left( x_{i}-\bar{X} \right)^{2}}} \right)}^{\frac{1}{2}}$$

The change in SD can therefore be written as

$$\Delta SD=\sqrt{S^{2}+\beta}-\sqrt{S^{2}}$$

With the difference between the two radicands collected into the new parameter $\beta$. Both $\Delta SD$ and $\Delta\bar{X}$ are dependent on $n$. They are large for small $n$, and vanish for large $n$:

$$\lim_{n\to\infty} \Delta\bar{X}\to0$$

$$\lim_{n\to\infty} \Delta SD\to0$$

The values for $\Delta SD$ and $\Delta\bar{X}$ plotted in Figure 7 have been calculated by assuming that the parameters (variance and mean) of the hypothetical new study data are identical to that of the reference data. The corresponding R-script is given below.
